# Gross failure rates and failure modes for a commercial AI‐based auto‐segmentation algorithm in head and neck cancer patients

**DOI:** 10.1002/acm2.14273

**Published:** 2024-01-23

**Authors:** Simon W. P. Temple, Carl G. Rowbottom

**Affiliations:** ^1^ Medical Physics Department The Clatterbridge Cancer Centre NHS Foundation Trust Liverpool UK; ^2^ Department of Physics University of Liverpool Liverpool UK

**Keywords:** auto‐segmentation, deep learning, failure modes

## Abstract

**Purpose:**

Artificial intelligence (AI) based commercial software can be used to automatically delineate organs at risk (OAR), with potential for efficiency savings in the radiotherapy treatment planning pathway, and reduction of inter‐ and intra‐observer variability. There has been little research investigating gross failure rates and failure modes of such systems.

**Method:**

50 head and neck (H&N) patient data sets with “gold standard” contours were compared to AI‐generated contours to produce expected mean and standard deviation values for the Dice Similarity Coefficient (DSC), for four common H&N OARs (brainstem, mandible, left and right parotid). An AI‐based commercial system was applied to 500 H&N patients. AI‐generated contours were compared to manual contours, outlined by an expert human, and a gross failure was set at three standard deviations below the expected mean DSC. Failures were inspected to assess reason for failure of the AI‐based system with failures relating to suboptimal manual contouring censored. True failures were classified into 4 sub‐types (setup position, anatomy, image artefacts and unknown).

**Results:**

There were 24 true failures of the AI‐based commercial software, a gross failure rate of 1.2%. Fifteen failures were due to patient anatomy, four were due to dental image artefacts, three were due to patient position and two were unknown. True failure rates by OAR were 0.4% (brainstem), 2.2% (mandible), 1.4% (left parotid) and 0.8% (right parotid).

**Conclusion:**

True failures of the AI‐based system were predominantly associated with a non‐standard element within the CT scan. It is likely that these non‐standard elements were the reason for the gross failure, and suggests that patient datasets used to train the AI model did not contain sufficient heterogeneity of data. Regardless of the reasons for failure, the true failure rate for the AI‐based system in the H&N region for the OARs investigated was low (∼1%).

## 1 INTRODUCTION

The accurate delineation of organs at risk (OAR) for modern radiotherapy treatment planning is clearly an important step in the radiation oncology workflow.^1^ When performed manually this process can be extremely time‐consuming,^2^ and is prone to inter and intra‐observer variability.^3^, ^4^ The availability of any system which is able to automatically and accurately perform this function is therefore likely to produce substantial quality and efficiency savings, particularly in the head & neck region.^5^, ^6^, ^7^, ^8^, ^9^

Early solutions to auto‐segmentation used atlas‐based methods,^10^ but the use of artificial intelligence (AI) based software for auto‐segmentation of OARs has become increasingly common in recent years. A review of auto‐segmentation literature from 2008 to 2020 demonstrated that a shift from atlas‐based methods to AI‐based methods began around 2016.^11^

More specifically, deep learning (DL), which is a subset of AI, forms the basis for these new auto‐segmentation techniques,^12^ and it has been suggested that use of this new technology means we have now entered the fourth generation of auto‐segmentation algorithm development.^2^ A 2022 review of auto‐segmentation techniques used in radiotherapy treatment planning concluded DL methods have the potential to transform the radiation oncology workflow by increasing efficiency and removing inter and intra‐observer variability.^13^

Training sets for deep learning‐based auto‐segmentation models can be significant compared to earlier atlas‐based methods, although the number depends on representativeness of the training data and can be reduced via the application of augmentation techniques such as geometrical transformations of original images.^11^

Systems that utilize deep learning are often referred to as “black box,” because it is not possible for users to understand their internal function and therefore not possible to predict their behavior. There is therefore a need for robust studies to evaluate performance before such systems are used clinically.^14^

The concept of “Explainable Machine Learning” has previously been described^15^ and suggests that it is often possible to use interpretable black box models. To date, this approach has not been utilized for AI auto‐segmentation in radiation oncology. Discussions of the use of black box AI in medicine more generally suggests that interpretability is a requirement to gain trust and acceptance of AI in medicine from physicians.^16^

The importance of auto‐segmentation system quality assurance has previously been stressed due to the potentially serious consequences of segmentation errors with end‐users advised to employ both case‐specific and routine model quality assurance on such systems.^2^, ^17^ Currently this is mainly achieved by manual inspection of any auto‐segmented contours generated for all patients due to a lack of knowledge of failure rates and likely failure modes from auto‐segmentation software.^18^

The aim of this study was to identify the failure rate and failure modes of a commercial AI‐based auto‐segmentation system generating head and neck (H&N) OARs. There has been little research investigating gross failure rates and failure modes of such systems.

## 2 METHODS

In order to be able to define a “gross failure” it is important to establish expected behavior. For auto‐segmentation expected behavior can be quantified using similarity metrics. In this study, the Dice Similarity Coefficient (DSC)^19^ was used to define normal range of similarity.

Initially, 50 anonymized H&N patient data sets with “gold standard” contours were compared to contours generated by an AI‐based system (Mirada DLC Expert™). Mirada^A^ DLC Expert™ is a commercially available AI‐based system for the generation of organs‐at‐risk (OARs) used in radiotherapy treatment planning. As previously described, the software uses multiple convolutional networks to learn features in the input images to generate a semantic segmentation. A coarse resolution OAR output from an initial 2D multi‐class network with 14 layers, along with the CT image data, is fed into a separately trained 10‐layer OAR‐specific network to predict full‐resolution contours.^20^, ^21^

A standard DLC Expert model, H&N CT NL004 GN, was used to generate the AI‐based contours with no local customization. The contours are based on published international contouring guidelines^22^ and contained a training dataset of 698 H&N patients with 2 mm CT slices, without contrast, and 512 × 512 in‐plane resolution. The model contains 22 OAR‐specific networks for full‐resolution output. Table 1 lists the OARs available in the model.

**TABLE 1:** OARs available in the DLC expert model (Model: H&N CT NL004 GN).

OAR Structure Name		
A_carotid_l	Cerebrum	Pharynxcost
A_carotid_r	Crico	Spinalcord
Arytenoid_l	Esophagus_cerv	Submandibular_l
Arytenoid_r	Gloticarea	Submandibuar_r
Brainstem	Mandible	Supgraglottic
Buccalmucosa_l	Oralcavity_ext	Thyroid
Buccalmucosa_r	Parotid_l	
Cerebellum	Parotid_r	

All manual contouring data originated from patients previously enrolled in the PATHOS clinical trial.^23^ This patient cohort was selected because the associated trial protocol included clear anatomical guidelines for OAR delineation and, in addition, trial entry involved pre‐trial OAR outlining quality assurance, which all Oncologists were required to undertake. Guidelines and teaching have previously been shown to significantly reduce inter‐observer contour variability.^24^ A sub‐sample of patient data was retrospectively reviewed during the study to provide further assurance around the quality of contours used. For the purpose of this research, the contours were deemed to be of “gold standard” when comparing to automatically generated contours. Mean and standard deviation values for the similarity metric, DSC, for four commonly used OARs in the H&N region (brainstem, mandible, left and right parotid) were established. This data was used to define the lower limit of DSC expected for each OAR.

The same commercial AI‐based system was then used to generate four commonly used OARs (brainstem, mandible, left and right parotid) on a further 500 anonymised patient CT data sets. A data set of this size was determined to be necessary due to the absence of any existing evidence on failure rates and the need to identify a sufficiently accurate failure rate. The 500 data sets also contained contours that had been previously generated manually, by a human expert. The AI‐based contours were compared to the manual contours using the Mirada Contour Insights™ tool to produce a 3D DSC for each patient.

Mirada DLC Expert accepts all patients without restriction on age, and can therefore be used for both adult and pediatric patients. For the 500 patient test sample used, 498 were adults, with 1 pediatric patient, aged 2, and 1 young adult, aged 23. The test sample contained a 72:28 male‐to‐female ratio and the median patient age was 63.

To identify gross failures of the AI‐generated contours, a three‐sigma limit was set to determine the failure rate, meaning that 99.7% of results can be assumed to be within this limit.^25^ All failures for each OAR were manually inspected by an expert observer, and reasons for failure, or failure mode, were categorized as shown in Table 2. Failures identified as due to suboptimal manual contouring were censored.

**TABLE 2:** Categorization of failures.

Category	Description
Setup	Non‐standard CT scan patient setup position
Anatomy	Non‐standard internal patient anatomy, e.g., post surgery
Artefacts	CT artefacts present in region due to, e.g., dental fillings
Unknown	No obvious reason for failure

## 3 RESULTS

Table 3 summarizes the mean and standard deviations of DSC for the four OARs from the initial 50‐patient cohort: brainstem, mandible, left parotid and right parotid.

**TABLE 3:** DSC values from the 50‐patient cohort study.

OAR (Mean – 3xSD)	DSC (±1SD)	Gross failure level
Brainstem	0.81 ± 0.06	0.63
Mandible	0.91 ± 0.02	0.85
Left parotid	0.76 ± 0.06	0.58
Right parotid	0.74 ± 0.08	0.51

**FIGURE 1:**
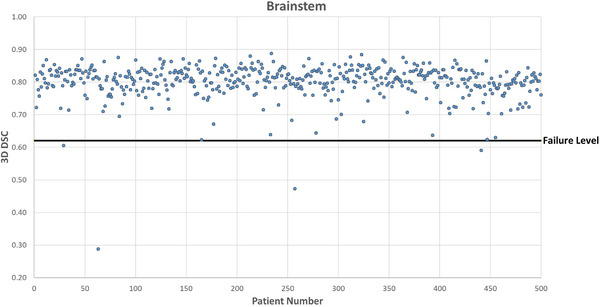
Brainstem OAR DSC for 500 patients. 3D DSC for the comparison between AI‐based auto‐segmented and manually delineated brainstem OAR.

**FIGURE 2:**
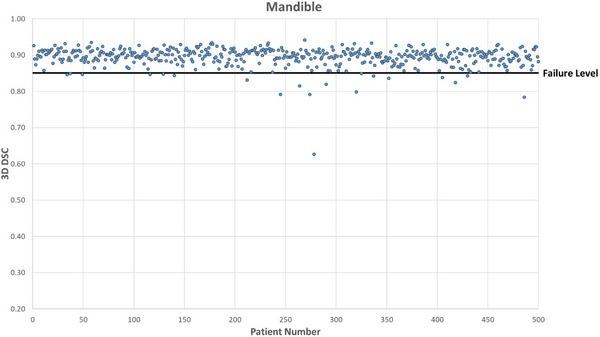
Mandible OAR DSC for 500 patients. 3D DSC for the comparison between AI‐based auto‐segmented and manually delineated mandible OAR.

**FIGURE 3:**
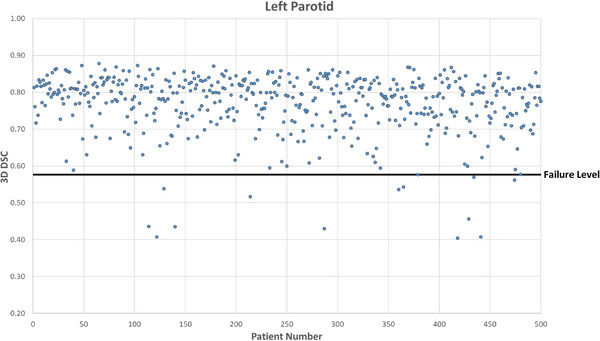
Left parotid OAR DSC for 500 patients. 3D DSC for the comparison between AI‐based auto‐segmented and manually delineated left parotid OAR.

**FIGURE 4:**
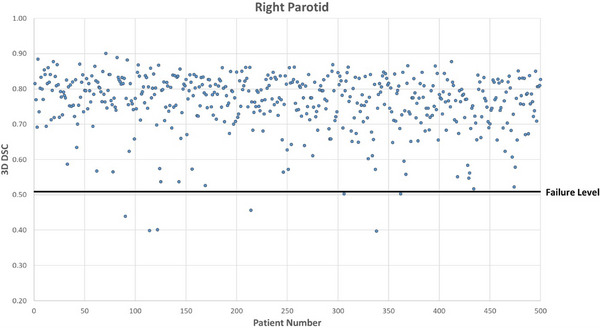
Right parotid OAR DSC for 500 patients. 3D DSC for the comparison between AI‐based auto‐segmented and manually delineated right parotid OAR.

Figures 1, 2, 3, 4 show DSC values for the comparison between AI auto‐segmented and manually delineated OARs for the 500 patient cohort. The failure level is set at three standard deviations below the expected mean DSC value. The overall mean failure rate for the four OARs investigated was found to be 1.2%.

Resulting failure rates by OAR are shown in Table 4. For the brainstem, there were 2 true failures and a true failure rate of 0.4%. The mandible structure had 11 true failures after censoring of suboptimal clinical contours. Of these 8 were due to unusual patient anatomy and 3 appeared to be caused by dental artefacts.

**TABLE 4:** AI‐based auto‐segmentation failure rates from 500 patient cohort.

	Brainstem	Mandible	Lt Parotid	Rt Parotid
**Failures**	4	20	13	7
Censored (sub‐optimal manual contour)	2	9	6	3
**Failure reason**:				
Setup position	2	0	0	1
Anatomical	0	8	5	2
Image artefacts	0	3	0	1
Unknown	0	0	2	0
**True failures**	**2**	**11**	**7**	**4**
**True failure rate**	**0.4%**	**2.2%**	**1.4%**	**0.8%**

For the left parotid there were 7 true failures. Reasons for failure were determined to be caused by unusual patient anatomy for 5 patients, and for the 2 remaining patients the failure reason could not be identified. For the right parotid there were 4 true failures. One failure was determined to be caused by a non‐standard patient setup position, 2 were due to unusual patient anatomy and 1 was caused by a dental artefact in the CT scan.

**FIGURE 5:**
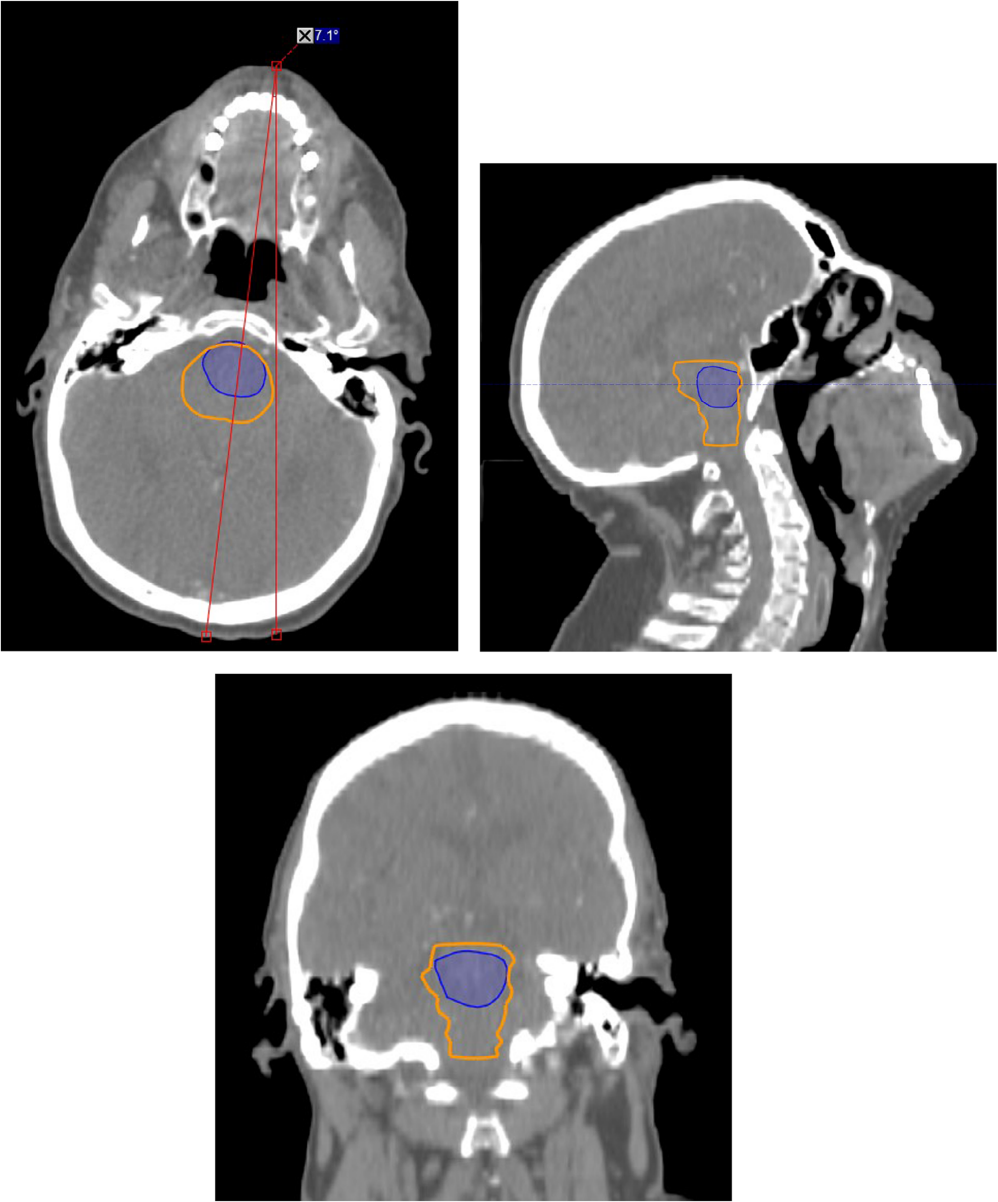
Failure due to setup. Axial, sagittal, and coronal CT images illustrating an example of a gross failure for auto‐segmentation of the brainstem. The AI‐based auto‐segmented contour is blue and the manual contour is orange.

An example of a setup failure for the brainstem OAR is shown in Figure 5. It can be observed that this patient had an obvious “roll” in their setup position. When measured, the axial roll was found to be approximately 7°.

**FIGURE 6:**
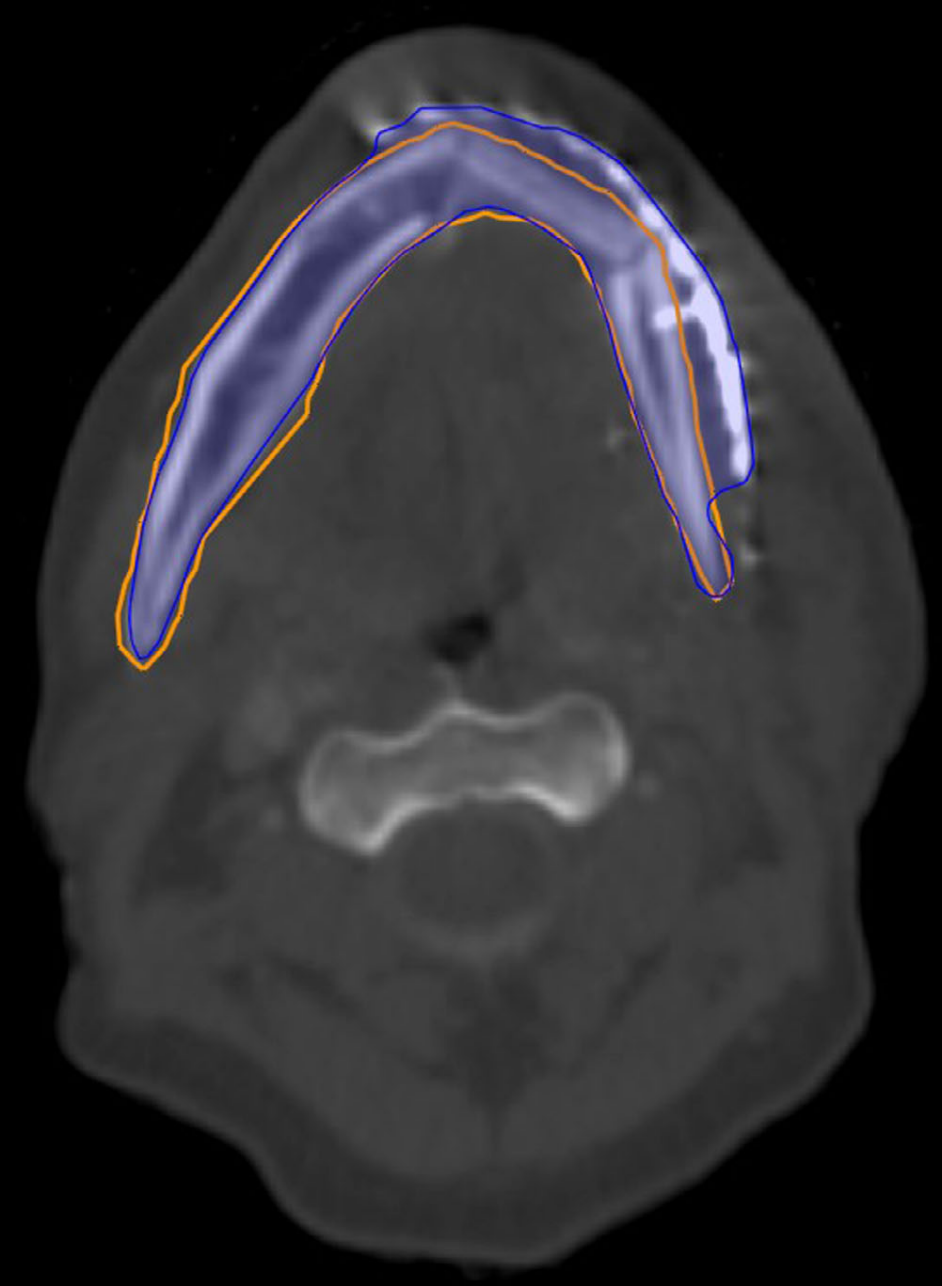
Failure due to anatomy. Axial CT image illustrating an example of a gross failure for auto‐segmentation of the mandible. The AI‐based auto‐segmented contour is blue and the manual contour is orange. The AI‐based auto‐segmented contour has included the surgical plate as well as the mandible.

An example of anatomical failure for the mandible OAR is shown in Figure 6. It can be observed that the auto‐segmented mandible contour includes a surgical metal plate.

**FIGURE 7:**
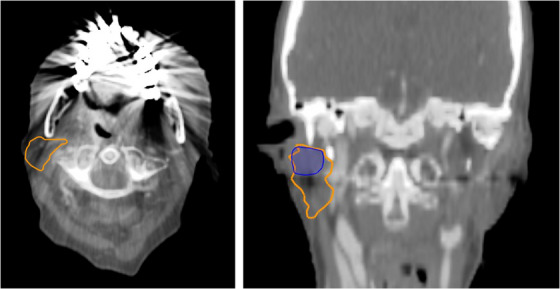
Failure due to artefact. Coronal and axial CT images illustrating an example of a gross failure for auto‐segmentation of the right parotid. The AI‐based auto‐segmented contour is blue and the manual contour is orange.

An example of failure for the right parotid OAR is shown in Figure 7. It can be observed that the inferior extent of the auto‐segmented parotid contour stops at the level where dental CT artefacts are present.

## 4 DISCUSSION

The aim of this study was to assess gross failure rates and identify any common failure modes of a commercial AI‐based auto‐segmentation system. The four OARs (brainstem, mandible, left and right parotid) were chosen as they were routinely, manually, outlined for all H&N patients at our center and could therefore be compared to AI‐based contours for a large retrospective patient cohort without the need for additional curation of the data. The spinal cord was also routinely contoured and initially considered but subsequently excluded from the study as the inferior border of the manually outlined spinal cord was not anatomically defined. This resulted in the length of spinal cord manually contoured varying significantly from patient to patient.

The introduction of AI‐based auto‐segmentation software often leads to more OARs being routinely outlined compared to traditional manual contouring. Failure rates for the additional OARs should be established as part of post‐implementation surveillance. This could be achieved via tracking of the number of major manual corrections required to the auto‐generated AI‐based contours for the first 500 instances of any new OAR being introduced. Given the low failure rates expected, a large sample size would be needed for additional OARs, in line with this study.

The 3D DSC metric was used in the study to identify gross failures of the AI‐based OAR contours. Spatial‐based metrics have been suggested as complimentary to volume‐based metrics when comparing auto‐generated contours with gold‐standard manual contours.^26^ The 2D 95% Hausdorff distance metric^27^ was also considered within the study, but was found not to be effective at reliably identifying gross failures of the AI‐based contouring system.

To 2D Hausdorff distance is calculated from 2 contours of the same OAR. For CT slices with only one contour for the OAR, the 2D Hausdorff distances cannot be calculated, and therefore the 95% 2D Hausdorff distance for the OAR will not fully represent the similarity of the 2 contours under consideration. Gross failures of the AI‐based system often involved generation of a substantially smaller OAR in the superior‐inferior direction compared to the gold standard contours. This can clearly be seen in Figures 5 and 7. Distance‐based metrics such as the 2D Hausdorff distance give incomplete values for these types of gross failures. For example, for the patient shown in Figure 5, the 2D 95% Hausdorff distance correctly identifies a gross error, even though it is only calculating the Hausdorff distances on the CT slices containing both manual and AI‐based contours. This is because on the CT slices containing both gold standard and AI‐based contours, there is a significant distance between the two contours resulting in a 2D 95% Hausdorff distance of 13.7 mm, larger than the upper limit threshold of 11.6 mm for this OAR. For the patient shown in Figure 7, the 2D 95% Hausdorff distance again only calculates the distances on the CT slices containing both the manual and AI‐based contours, but in this case, does not identify a gross error. In this case, the manual and AI‐based contours are similar in size, shape and position resulting in a 2D 95% Hausdorff distance of 7.6 mm against an upper limit threshold of 22.8 mm for this OAR.

By contrast, the patient shown in Figure 6, where a surgical plate was incorrectly included in the AI‐based contour, the superior‐inferior length of the contour was the same as the gold standard manual contour resulting in an accurate evaluation of the 2D 95% Hausdorff distance that exceeded the threshold for the mandible and correctly identified a gross error in this case. The three examples demonstrate the limitations and potential drawbacks of using a 2D‐based distance metric when considering gross differences between multiple contours.

The study has shown that true gross failures of such a system is rare, being less than 1% for some OARs, for example, brainstem, and an overall mean failure rate of 1.2% for all OARs. One of the reasons for failure, the presence of dental artefacts, is both easy to identify on CT scans as a possible issue during review, and less likely to apply to modern CT scanners, which utilise metal artefact reduction algorithms.^28^

In terms of the other failure modes identified, patient setup position and non‐standard anatomy, it is likely that such failures are caused by the absence of sufficient numbers of these “unusual” case types in the model training data set of the AI‐based auto‐segmentation software. This sort of dataset bias in AI is a well‐known^29^, ^30^, ^31^, ^32^, ^33^, ^34^ and should be expected, given the relative frequency of such cases in the clinic. In addition, due to the observed wide anatomical variation in patients with non‐standard anatomy, it may not be possible to include sufficient numbers of this patient type in a model training data set for the training to be sufficiently effective due to the large patient dataset numbers typically required to train DL models.^35^

These findings highlight the need for manufacturers to “open up” the black box nature of the DL software being produced. More specifically, datasheets containing comprehensive information about the dataset used to produce a DL model should be provided,^36^ and such information could be used by healthcare professionals to guide the limits of clinical use and to devise appropriately targeted quality assurance methods.

Regardless of the reasons for failure, due to the extremely low true failure rates identified, the ideal approach for the QA of auto‐segmented OARs would be to use stratified methods, rather than 100% manual human expert inspection. This is partly because attribute inspection errors by humans will always exist,^37^ and with errors occurring at such low rates it is a distinct possibility that they may be missed by a human who does not often encounter such errors, or that multiple checks would be required to provide sufficient levels of safety,^38^ which would add an increased quality assurance burden for a process with very small failure rates.

It is important to note that this research is looking at gross failures, rather than more subtle quality issues, which may still be clinically significant. Recent research has shown that the quality of contours that are being produced by some AI auto‐segmentation systems are not yet at the level where they do not sometimes require further manual editing.^39^, ^40^, ^41^ It should, however, be noted that human observer variation can also be of a clinically significant magnitude,^42^ yet such differences are often not considered significant when a human is involved. For example, a previous study^43^ found mandible inter‐observer variability to have a median value of 0.9 which is a similar order of magnitude to the results obtained in this study and suggests that the quality of some AI‐based auto‐segmented OARs may already have reached human expert levels. This is supported by a separate study which concluded that the accuracy of AI auto‐segmented contours is now at a comparable level to that of expert inter‐observer variability.^14^

Future research to assess the clinical significance of minor contouring failures would therefore be beneficial to determine the true importance of the often‐perceived requirement for further human manual inspection of contours produced by modern commercial AI auto‐segmentation systems.

A further point of interest is that suboptimal clinical contours made up 45.5% of initial total failures in this study using real world data. This failure rate is a similar order of magnitude to the true failure rate of the AI‐based system, and raises questions around clinical significance of these failures which could be investigated in future research.

## 5 CONCLUSION

To conclude, this study has demonstrated that gross failure rates for the H&N OARs tested, using a modern commercial AI‐based auto‐segmentation system, are extremely low.

It is also recommended that manufacturers provide greater information relating to the datasets they use to produce AI models to assist users with identifying potential dataset bias, and that manufacturers attempt to further reduce this bias in future models.

Recent research has shown that differences between gold standard and AI‐based auto‐segmented contours are at a comparable level to inter and intra‐observer variability differences. It is therefore suggested that as resulting auto‐segmented contour quality improves with future iterations of this technology, it may be possible to remove the need for 100% manual human expert inspection in the near future. This approach would require sufficiently accurate quality assurance methods to be included as part of the workflow.

## AUTHOR CONTRIBUTIONS

Simon Temple contributed to study design, data collection, analysis and interpretation, and drafting of the manuscript. Carl Rowbottom contributed to study design, data collection, analysis and interpretation, and drafting of the manuscript.

## CONFLICT OF INTEREST STATEMENT

The authors declare no conflicts of interest.

## Footnotes

A Mirada Medical Systems Ltd, Oxford. UK.

## References

[1] Tong N , Gou S , Yang S , Ruan D , Sheng K . Fully automatic multi‐organ segmentation for head and neck cancer radiotherapy using shape representation model constrained fully convolutional neural networks. Med Phys. 2018;45(10):4558‐4567. doi:10.1002/mp.13147 30136285 PMC6181786

[2] Cardenas CE , Yang J , Anderson BM , Court LE , Brock KB . Advances in auto‐segmentation. Semin Radiat Oncol. 2019;29(3):185‐197. doi:10.1016/J.SEMRADONC.2019.02.001 31027636

[3] Nelms BE , Robinson G , Markham J , et al. Variation in external beam treatment plan quality: an inter‐institutional study of planners and planning systems. Pract Radiat Oncol. 2012;2(4):296‐305. doi:10.1016/j.prro.2011.11.012 24674168

[4] Stelmes JJ , Vu E , Grégoire V , et al. Quality assurance of radiotherapy in the ongoing EORTC 1420 “Best of” trial for early stage oropharyngeal, supraglottic and hypopharyngeal carcinoma: results of the benchmark case procedure. Radiat Oncol. 2021;16(1):1‐10. doi:10.1186/s13014-021-01809-2 33933118 PMC8088557

[5] van der Veen J , Willems S , Deschuymer S , et al. Benefits of deep learning for delineation of organs at risk in head and neck cancer. Radiother Oncol. 2019;138:68‐74. doi:10.1016/J.RADONC.2019.05.010 31146073

[6] Urago Y , Okamoto H , Kaneda T , et al. Evaluation of auto‐segmentation accuracy of cloud‐based artificial intelligence and atlas‐based models. Radiat Oncol. 2021;16(1):175. doi:10.1186/s13014-021-01896-1 34503533 PMC8427857

[7] Walker Z , Bartley G , Hague C , et al. Evaluating the effectiveness of deep learning contouring across multiple radiotherapy centres. Phys imaging Radiat Oncol. 2022;24:121‐128. doi:10.1016/j.phro.2022.11.003 36405563 PMC9668733

[8] Hu Y , Nguyen H , Smith C , et al. Clinical assessment of a novel machine‐learning automated contouring tool for radiotherapy planning. J Appl Clin Med Phys. 2023;24(7):e13949. doi:10.1002/acm2.13949 36871161 PMC10338747

[9] Lucido JJ , DeWees TA , Leavitt TR , et al. Validation of clinical acceptability of deep‐learning‐based automated segmentation of organs‐at‐risk for head‐and‐neck radiotherapy treatment planning. Front Oncol. 2023;13:1137803. doi:10.3389/fonc.2023.1137803 37091160 PMC10115982

[10] Daisne J‐F , Blumhofer A . Atlas‐based automatic segmentation of head and neck organs at risk and nodal target volumes: a clinical validation. Radiat Oncol. 2013;8:154. doi:10.1186/1748-717X-8-154 23803232 PMC3722083

[11] Vrtovec T , Močnik D , Strojan P , Pernuš F , Ibragimov B . Auto‐segmentation of organs at risk for head and neck radiotherapy planning: from atlas‐based to deep learning methods. Med Phys. 2020;47(9):e929‐e950. doi:10.1002/mp.14320 32510603

[12] Samarasinghe G , Jameson M , Vinod S , et al. Deep learning for segmentation in radiation therapy planning: a review. J Med Imaging Radiat Oncol. 2021;65(5):578‐595. doi:10.1111/1754-9485.13286 34313006

[13] Harrison K , Pullen H , Welsh C , Oktay O , Alvarez‐Valle J , Jena R . Machine learning for auto‐segmentation in radiotherapy planning. Clin Oncol (R Coll Radiol). 2022;34(2):74‐88. doi:10.1016/j.clon.2021.12.003 34996682

[14] Wong J , Fong A , McVicar N , et al. Comparing deep learning‐based auto‐segmentation of organs at risk and clinical target volumes to expert inter‐observer variability in radiotherapy planning. Radiother Oncol. 2020;144:152‐158. doi:10.1016/J.RADONC.2019.10.019 31812930

[15] Rudin C , Radin J . Why are we using black box models in ai when we don't need to? A lesson from an explainable AI competition. Harvard Data Sci Rev. 2019;1(2):1‐10. doi:10.1162/99608f92.5a8a3a3d

[16] Poon AIF , Sung JJY . Opening the black box of AI‐Medicine. J Gastroenterol Hepatol. 2021;36(3):581‐584. doi:10.1111/jgh.15384 33709609

[17] Vandewinckele L , Claessens M , Dinkla A , et al. Overview of artificial intelligence‐based applications in radiotherapy: recommendations for implementation and quality assurance. Radiother Oncol. 2020;153:55‐66. doi:10.1016/j.radonc.2020.09.008 32920005

[18] Brouwer CL , Dinkla AM , Vandewinckele L , et al. Machine learning applications in radiation oncology: current use and needs to support clinical implementation. Phys Imaging Radiat Oncol. 2020;16(November):144‐148. doi:10.1016/j.phro.2020.11.002 33458358 PMC7807598

[19] Dice LR . Measures of the amount of ecologic association between species. Ecology. 1945;26(3):297‐302. doi:10.2307/1932409

[20] Yang J , Veeraraghavan H , Armato SG , et al. Autosegmentation for thoracic radiation treatment planning: a grand challenge at AAPM 2017. Medical Physics. 2018;45(10):4568‐4581. doi:10.1002/mp.13141 30144101 PMC6714977

[21] van Dijk LV , Van den Bosch L , Aljabar P , et al. Improving automatic delineation for head and neck organs at risk by deep learning contouring. Radiother Oncol . 2020;142:115‐123. doi:10.1016/j.radonc.2019.09.022 31653573

[22] Brouwer CL , Steenbakkers RJHM , Bourhis J , et al. CT‐based delineation of organs at risk in the head and neck region: DAHANCA, EORTC, GORTEC, HKNPCSG, NCIC CTG, NCRI, NRG Oncology and TROG consensus guidelines. Radiother Oncol . 2015;117(1):83‐90. doi:10.1016/j.radonc.2015.07.041 26277855

[23] Owadally W , Hurt C , Timmins H , et al. PATHOS: a phase II/III trial of risk‐stratified, reduced intensity adjuvant treatment in patients undergoing transoral surgery for Human papillomavirus (HPV) positive oropharyngeal cancer. BMC Cancer. 2015;15(1):1‐10. doi:10.1186/s12885-015-1598-x 26311526 PMC4549836

[24] Vinod SK , Min M , Jameson MG , Holloway LC . A review of interventions to reduce inter‐observer variability in volume delineation in radiation oncology. J Med Imaging Radiat Oncol. 2016;60(3):393‐406. doi:10.1111/1754-9485.12462 27170216

[25] Pukelsheim F . The three sigma rule. Am Stat . 1994;48(2):88‐91. doi:10.1080/00031305.1994.10476030

[26] Sherer MV , Lin D , Elguindi S , et al. Metrics to evaluate the performance of auto‐segmentation for radiation treatment planning: a critical review. Radiother Oncol . 2021;160:185‐191. doi:10.1016/j.radonc.2021.05.003 33984348 PMC9444281

[27] Huger S , Graff P , Harter V , Marchesi V , et al. Evaluation of the Block Matching deformable registration algorithm in the field of head‐and‐neck adaptive radiotherapy. Physica Medica. 2014;30(3):301‐308. doi:10.1016/j.ejmp.2013.09.001 24090743

[28] Kovacs DG , Rechner LA , Appelt AL , et al. Metal artefact reduction for accurate tumour delineation in radiotherapy. Radiother Oncol. 2018;126(3):479‐486. doi:10.1016/j.radonc.2017.09.029 29050958 PMC5864514

[29] Kusters R , Misevic D , Berry H , et al. Interdisciplinary research in artificial intelligence: challenges and opportunities. Front big data. 2020;3:577974. doi:10.3389/fdata.2020.577974 33693418 PMC7931862

[30] Alowais SA , Alghamdi SS , Alsuhebany N , et al. Revolutionizing healthcare: the role of artificial intelligence in clinical practice. BMC Med Educ. 2023;23(1):1‐15. doi:10.1186/s12909-023-04698-z 37740191 PMC10517477

[31] El Naqa I , Karolak A , Luo Y , et al. Translation of AI into oncology clinical practice. Oncogene. 2023;42:3089‐3097. doi:10.1038/s41388-023-02826-z 37684407 PMC12516697

[32] Filippi CG , Stein JM , Wang Z , et al. Ethical considerations and fairness in the use of artificial intelligence for neuroradiology. Am J Neuroradiol. 2023;44:1242‐1248. doi:10.3174/ajnr.a7963. Published online. 37652578 PMC10631523

[33] Jeyaraman M , Balaji S , Jeyaraman N , Yadav S . Unraveling the ethical enigma: artificial intelligence in healthcare. Cureus. 2023;15(8):8‐13. doi:10.7759/cureus.43262 PMC10492220 37692617

[34] Polevikov S . Advancing AI in healthcare: a comprehensive review of best practices. Clin Chim Acta. 2023;548(July):117519. doi:10.1016/j.cca.2023.117519 37595864

[35] Fan J , Wang J , Chen Z , Hu C , Zhang Z , Hu W . Automatic treatment planning based on three‐dimensional dose distribution predicted from deep learning technique. Med Phys. 2019;46(1):370‐381. doi:10.1002/mp.13271 30383300

[36] Gebru T , Morgenstern J , Vecchione B , et al. Datasheets for datasets. Commun ACM. 2021;64(12):86‐92. doi:10.1145/3458723

[37] Burke R . Inspection planning for mission‐critical quality. IEEE Int Eng Manag Conf. 2001:329‐334. doi:10.1109/iemc.2001.960553. Published online.

[38] Papadakis EP , Stephan CH , McGinty MT , Wall WB . Inspection decision theory: deming inspection criterion and time‐adjusted rate‐of‐return compared. Eng Costs Prod Econ. 1988;13(2):111‐124. doi:10.1016/0167-188X(88)90025-0

[39] Rhee DJ , Cardenas CE , Elhalawani H , et al. Automatic detection of contouring errors using convolutional neural networks. Med Phys. 2019;46(11):5086‐5097. doi:10.1002/MP.13814 31505046 PMC6842055

[40] Robert C , Munoz A , Moreau D , et al. Clinical implementation of deep‐learning based auto‐contouring tools–Experience of three French radiotherapy centers. Cancer/Radiotherapie. 2021;25(6‐7):607‐616. doi:10.1016/J.CANRAD.2021.06.023 34389243

[41] Brouwer CL , Boukerroui D , Oliveira J , et al. Assessment of manual adjustment performed in clinical practice following deep learning contouring for head and neck organs at risk in radiotherapy. Phys imaging Radiat Oncol. 2020;16:54‐60. doi:10.1016/j.phro.2020.10.001 33458344 PMC7807591

[42] Peng Y‐L , Chen L , Shen G‐Z , et al. Interobserver variations in the delineation of target volumes and organs at risk and their impact on dose distribution in intensity‐modulated radiation therapy for nasopharyngeal carcinoma. Oral Oncol. 2018;82:1‐7. doi:10.1016/j.oraloncology.2018.04.025 29909882

[43] van der Veen J , Gulyban A , Willems S , Maes F , Nuyts S . Interobserver variability in organ at risk delineation in head and neck cancer. Radiat Oncol. 2021;16(1):1‐12. doi:10.1186/s13014-020-01677-2 34183040 PMC8240214

